# S-Propargyl-Cysteine Remodels the Gut Microbiota to Alleviate Rheumatoid Arthritis by Regulating Bile Acid Metabolism

**DOI:** 10.3389/fcimb.2021.670593

**Published:** 2021-08-06

**Authors:** Zhou Wang, Yue Yu, Junyi Liao, Wei Hu, Xiqing Bian, Jianlin Wu, Yi Zhun Zhu

**Affiliations:** ^1^State Key Laboratory of Quality Research in Chinese Medicine & School of Pharmacy, Macau University of Science and Technology, Macau, Macau; ^2^Shanghai Key Laboratory of Bioactive Small Molecules & School of Pharmacy, Fudan University, Shanghai, China

**Keywords:** S-propargyl-cysteine, gut microbiota, rheumatoid arthritis, bile acids metabolism, 16S rRNA

## Abstract

**Background:**

Rheumatoid arthritis (RA) is a long-term autoimmune disorder characterized by chronic inflammation that results in swollen and painful joints and even cartilage and bone damage. The gut microbiota, a novel anti-inflammatory target, is considered an important environmental factor in the development of RA. S-propargyl-cysteine (SPRC), an amino acid analogue, exerts anti-inflammatory, cardioprotective effects, and neuroprotective effects on various diseases. In recent studies, an SPRC treatment exerted anti-inflammatory effects on RA. Meanwhile, gut microbiome dysbiosis in individuals with RA has also been reported by many researchers. However, the relationship between SPRC and gut microbiota in individuals with RA remains unclear.

**Methods:**

Thirty male Sprague-Dawley (SD) rats were randomly divided into three groups of 10 each, including the Control, Model, and SPRC groups. Adjuvant-induced arthritis (AIA) rats in SPRC group were treated with SPRC. Measurement of paw volume and serum tumor necrosis factor-α (TNF-α) and interleukin 6 (IL-6) levels were applied to evaluate the inflammatory status. Fecal samples were collected on the 14^th^ day and 28^th^ day. Gut microbiota were analyzed using 16S ribosomal RNA (rRNA) gene amplicon sequencing. Untargeted metabolomics on plasma samples was applied to investigate the metabolic changes induced by the altered gut microbiota by using derivatization-UHPLC-Q-TOF/MS.

**Findings:**

Using 16S rRNA amplicon sequencing, we found that SPRC significantly altered the gut microbiota structure in AIA rats. In particular, *Bifidobacterium*, a genus of BSH (Bile Salt Hydrolase)-producing microbes, was overrepresented in SPRC-treated AIA rats. Additionally, a subsequent metabolomics analysis indicated that bile acid metabolism was also altered by SPRC treatment. Interestingly, glycochenodeoxycholic acid (GCDCA) and glycocholic acid (GCA), which are formed with the participation of BSH-producing microbes in the intestine, were identified as crucial biomarkers responding to SPRC treatment with significantly lowered levels.

**Interpretation:**

A mechanistic link between the gut microbiota and plasma metabolites was revealed in this study, which provides insights into the mechanism of SPRC treatment for RA from the perspective of the gut microbiota.

## Introduction

Rheumatoid arthritis (RA) is a chronic inflammatory disease that can lead to cartilage and bone damage and even disability (Smolen et al., 2016). RA affects tens of millions of people and is associated with an increasing mortality rate due to cardiovascular and other systemic complications (England et al., 2018). In the past few decades, the treatment of RA has evolved substantially with the development of new classification criteria and the introduction of novel therapies (Stoffer et al., 2016). Currently, with early diagnosis and proper treatment, many patients with RA can achieve the current therapeutic goal, namely, remission or at least low disease activity (Burmester and Pope, 2017). However, a large number of patients fail to achieve this goal, implying urgent needs for novel therapeutic strategies.

The gut microbiota plays a fundamental role in shaping the immune response and immune metabolism. Moreover, it has also been considered an important factor in the development of RA (Honda and Littman, 2012; Valdes et al., 2018; du Teil Espina et al., 2019). According to previous research, dysbiosis is detected in the gut microbiota of patients with RA and is potentially resolved after treatment (Zhang et al., 2015). Alterations in gut microbiota are also correlated closely with clinical measurements, and some specific phylotypes have subsequently been identified as potential biomarkers for the early diagnosis of many inflammatory diseases (Maeda and Takeda, 2017). With an emerging understanding of the relationship between the gut microbiota and the immune system, increasing interest is now focused on microbiota-based therapeutic management of RA (Marietta et al., 2016; de Oliveira et al., 2017; Jeong et al., 2018).

S-propargyl-cysteine (SPRC), a novel amino acid analogue, is an endogenous hydrogen sulfide (H_2_S) donor that provides a substrate for H_2_S synthesis. As an important gasotransmitter, H_2_S is widely accepted as an anti-inflammatory mediator in various conditions (Burguera et al., 2017; Powell et al., 2018). According to previous studies, SPRC exerts an anti-inflammatory effect on many inflammatory diseases, such as cardiovascular diseases and neurodegenerative diseases (Wen and Zhu, 2015; Wen et al., 2018). Moreover, SPRC was also revealed to be an effective RA treatment. Wu et al. (2016) suggested that SPRC attenuates the inflammatory response in the MH7A cell line and in adjuvant-induced arthritis (AIA) rats by regulating the Nrf2-antioxidant response element pathway. Although several mechanistic links between SPRC treatment and inflammatory disease have already been elucidated, limited information is available about why its anti-inflammatory effect has a low risk of side effects (Smallwood et al., 2018).

In this study, we investigated the relationship between the gut microbiota and RA progression in SPRC-treated AIA rats and determined changes in the levels of valuable biomarkers in response to SPRC treatment. Untargeted metabolomics was also applied to investigate the metabolic changes induced by the altered gut microbiota, and a causal mechanism was further revealed between bile acid metabolism and gut microbiota in SPRC treatment.

## Materials and Methods

### Chemical Materials

SPRC was synthesized as reported in our previous study and purified by recrystallization from ethanol-water mixture (99%). Complete Freund’s adjuvant (CFA), acetonitrile, formic acid, and isopropanol were obtained from MultiSciences (Hangzhou, China). Reagents used in the gut microbiota experiment were provided by Sangon Biotech (Shanghai, China).

### Animals

The Animal Care and Use Committee of Municipal Affairs Bureau of Macau approved all studies described here (approval number AL010/DICV/SIS/2018), and the experiment was conducted according to the NIH Guide for the Care and Use of Laboratory Animals (8^th^ edition).

Thirty male Sprague-Dawley (SD) rats weighing 200–230 g were randomly divided into three groups of 10 each, including the Control, Model, and SPRC groups. Rats in the Model group and SPRC group both received a subcutaneous injection of 100 μl of CFA (10 mg·ml^−1^) at the base of the tail to establish the AIA model, while the rats in the Control group were injected with the same volume of saline. After injection, rats in the SPRC group were administered SPRC (100 mg·kg^-1^, as described in our previous study (Wu et al., 2016)) by intragastric administration once per day, while the rats in both the Control and Model groups were administered saline. Body weight was measured on the 0, 5^th^, 15^th^, 20^th^, and 25^th^ days after the CFA injection. All animals were sacrificed on the 28^th^ day, and blood samples were collected and stored at −80°C until use. Fecal samples were collected on the 14^th^ day and 28^th^ day after the CFA injection.

### Arthritis Index and Paw Volume Measurements

According to joint inflammation severity, the arthritis index was scored from 0 to 4 per limb, with a maximum score of 16 per animal, where 0 represented no signs of inflammation and 1–4 represented an increasing severity of inflammation (4 represented the most severe inflammation). The paw volume was measured using an Ugo Basile 7140 plethysmometer (Ugo Basile, Gemonio VA, Italy). Both the paw volume and arthritis index were recorded every 5 days after the CFA injection.

### Measurement of Serum Tumor Necrosis Factor-α and Interleukin 6 Levels Using Enzyme-Linked Immunosorbent Assays

Plasma samples obtained from the rats after sacrifice were analyzed for the concentrations of TNF-α and IL-6 using commercially available ELISA kits (Boatman Biotechnology, Shanghai, China) according to the manufacturer’s instructions. Optical densities were acquired with a microplate reader (M1000, TECAN, Austria GmbH, Austria) at 450 nm, and the results are presented as pg·ml^−1^.

### DNA Extraction

Total community genomic DNA extraction was performed using an E.Z.N.A. Soil DNA Kit (Omega, USA) according to the manufacturer’s instructions. The concentration of the DNA was measured using a Qubit 2.0 instrument (Life, USA) to ensure that adequate amounts of high-quality genomic DNA had been extracted.

### 16S rRNA Gene Amplification Using Polymerase Chain Reaction

The V3–V4 hypervariable region of the bacterial 16S rRNA gene was targeted. PCR was started immediately after the DNA was extracted. The 16S rRNA V3–V4 amplicon was amplified using KAPA HiFi Hot Start Ready Mix (2×) (TaKaRa Bio Inc., Japan). Two universal bacterial 16S rRNA gene amplicon PCR primers (PAGE purified) were used: the amplicon PCR forward primer (CCTACGGGNGGCWGCAG) and amplicon PCR reverse primer (GACTACHVGGGTATCTAATCC). The reaction was set up as follows: 2 μl of microbial DNA (10 ng/μl), 1 μl of amplicon PCR forward primer (10 μM), 1 μl of amplicon PCR reverse primer (10 μM), and 15 μl of 2× KAPA HiFi Hot Start Ready Mix. The plate was sealed, and PCR was performed in a thermal instrument (Applied Biosystems 9700, USA) using the following program: one cycle of denaturation at 95°C for 3 min, which included the first five cycles of denaturation at 95°C for 30 s, annealing at 45°C for 30 s, and elongation at 72°C for 30 s; 20 cycles of denaturation at 95°C for 30 s, annealing at 55°C for 30 s, and elongation at 72°C for 30 s; and a final extension at 72°C for 5 min. The PCR products were analyzed through electrophoresis on 1% (w/v) agarose gels in TBE buffer (Tris, boric acid, EDTA), stained with ethidium bromide (EB), and visualized under UV light.

### 16S Gene Library Construction, Quantification, and Sequencing

AMPure XP beads were used to purify the free primers and primer dimer species in the amplicon product. Samples were delivered to Sangon BioTech (Shanghai) for library construction using a universal Illumina adaptor and index. Before sequencing, the DNA concentration of each PCR product was determined using Qubit^®^ 2.0 Green double-stranded DNA assay, and it was quality-controlled using a bioanalyser (Agilent 2100, USA). Depending on coverage needs, all libraries can be pooled for one run. The amplicons from each reaction mixture were pooled in equimolar ratios based on their concentration. Sequencing was performed using the Illumina MiSeq system (Illumina MiSeq, USA) according to the manufacturer’s instructions.

### Sequence Processing

After sequencing, data were collected using the procedures described below (1). The two short Illumina reads were assembled by PEAR (v0.9.6) software according to the overlap, and fastq files were processed to generate individual fasta and qual files, which were then analyzed using standard methods. (2) Sequences containing ambiguous bases and any sequences longer than 480 base pairs (bp) were dislodged, and those with a maximum homopolymer length of 6 bp were allowed (Kochling et al., 2015), while those with sequences shorter than 200 bp were removed. (3) All identical sequences were merged into one. (4) Sequences were aligned according to a customized reference database. (5) The completeness of the index and the adaptor was checked, and all the index and the adaptor sequences were removed. (6) Noise was removed using the Pre.cluster tool. Chimeras were detected using Chimera UCHIME. All software was available in the mothur package. The effective sequences of each sample were submitted to the RDP Classifier again to identify archaeal and bacterial sequences. The modified pipeline was described on the mothur website. Finally, all effective bacterial sequences without primers were submitted for downstream analysis (Kozich et al., 2013).

### Bacterial Diversity and Taxonomic Analysis

Bacterial diversity and richness were determined by performing a sampling-based operational taxonomic unit (OTU) analysis and presented as the Chao1 index, ACE index, Shannon index, and Simpson index, which were calculated using the R program package “vegan”. Bacterial taxonomic analyses and comparisons at the bacterial phylum level were conducted between groups using the Wilcoxon rank sum test. Stool microbial characterization was subjected to linear discriminant analysis (LDA) effect size (LEfSe).

### PICRUSt Analysis

PICRUSt uses the OTU table accompanied by the relative abundance of samples as input to estimate the functional profile of the examined samples based on the known sequenced genomes (Douglas et al., 2018). The PICRUSt analysis was conducted using the website (http://picrust.github.io/).

### Metabolite Extraction

Fifty milligrams of blood samples were accurately weighed, and the metabolites were extracted using a 400 µl methanol:water (4:1, v/v) solution with 0.02 mg·ml L^−1^ 2-chlorophenylalanin as an internal standard. The mixture was allowed to settle at −10°C and treated with a Wonbio-96c high-throughput tissue crusher (Shanghai Wanbo Biotechnology Co., Ltd.) at 50 Hz for 6 min, followed by ultrasound at 40 kHz for 30 min at 5°C. The samples were placed at −20°C for 30 min to precipitate proteins. After centrifugation at 13,000 g for 15 min at 4°C, the supernatant was carefully transferred to sample vials for UHPLC-MS/MS analysis.

### Quality Control Sample

As a part of the system conditioning and quality control process, a pooled quality control sample (QC) was prepared by mixing equal volumes of all samples and treated in the same manner as the analytic samples. QC samples helped to represent the whole sample set, which would be injected at regular intervals (every 10 samples) to monitor the stability of the analysis.

### UHPLC-MS/MS Analysis of the Metabolites

Chromatographic separation of the metabolites was performed on a Thermo UHPLC system equipped with an ACQUITY UPLC HSS T3 column (100 mm × 2.1 mm i.d., 1.8 µm; Waters, Milford, USA).

The mobile phases consisted of 0.1% formic acid in water:acetonitrile (95:5, v/v) (solvent A) and 0.1% formic acid in acetonitrile:isopropanol:water (47.5:47.5:5, v/v) (solvent B). The solvent gradient changed according to the following conditions: 0 to 3.5 min, 0% B to 24.5% B (0.4 ml·min^-1^); 3.5 to 5 min, 24.5% B to 65% B (0.4 ml·min^−1^); 5 to 5.5 min, 65% B to 100% B (0.4 ml·min^-1^); 5.5 to 7.4 min, 100% B to 100% B (0.4 ml/min to 0.6 ml·min^−1^); 7.4 to 7.6 min, 100% B to 51.5% B (0.6 ml·min^−1^); 7.6 to 7.8 min, 51.5% B to 0% B (0.6 ml·min^−1^ to 0.5 ml·min^−1^); 7.8 to 9 min, 0% B to 0% B (0.5 ml·min^−1^ to 0.4 ml·min^−1^); and 9 to 10 min, 0% B to 0% B (0.4 ml·min^−1^) to equilibrate the systems. The injection volume was 2 µl, and the flow rate was set to 0.4 ml/min. The column temperature was maintained at 40°C. During the period of analysis, all these samples were stored at 4°C.

The mass spectrometry data were collected using a Thermo UHPLC-Q Exactive HF-X Mass Spectrometer equipped with an electrospray ionization (ESI) source operating in either positive or negative ion mode. The following optimal conditions were set: heater temperature, 425°C; capillary temperature, 325°C; sheath gas flow rate, 50 arb; aux gas flow rate, 13 arb; ion-spray voltage floating (ISVF), −3,500 V in negative mode and 3,500 V in positive mode; and normalized collision energy, 20-40-60 V rolling for MS/MS. The full MS resolution was 60,000, and the MS/MS resolution was 7,500. Data acquisition was performed with the Data Dependent Acquisition (DDA) mode. The detection was carried out over a mass range of 70–1,050 m/z.

### Data Preprocessing and Annotation

After UPLC-MS/MS analyses, the raw data were imported into Progenesis QI 2.3 (Nonlinear Dynamics, Waters, USA) for peak detection and alignment. The preprocessing results generated a data matrix that consisted of the retention time (RT), mass-to-charge ratio (m/z) values, and peak intensity. Metabolic features detected in at least 80% of any set of samples were retained. After filtering, minimum metabolite values were imputed for specific samples in which the metabolite levels fell below the lower limit of quantitation, and each metabolic feature was normalized to the sum. The internal standard was used for data QC (reproducibility). Metabolic features with a relative standard deviation (RSD) of QC>30% were discarded. Following normalization procedures and imputation, a statistical analysis was performed on log transformed data to identify significant differences in metabolite levels between comparable groups. Mass spectra of these metabolic features were identified based on the accurate mass, MS/MS fragment spectra, and isotope ratio differences and by searching reliable biochemical databases, such as the Human Metabolome Database (HMDB) (http://www.hmdb.ca/) and Metlin database (https://metlin.scripps.edu/). Specifically, the mass tolerance between the measured m/z values and the exact mass of the components of interest was ± 10 ppm. For metabolites with MS/MS confirmation, only those with MS/MS fragment scores >30 were considered confidently identified. Otherwise, metabolites had only tentative assignments.

### Statistical Analysis

Statistical analyses of the gut microbiome samples were performed using SPSS and GraphPad Prism (version 6.0) software packages. An unpaired Mann–Whitney rank sum test (two-tailed) was used for comparisons of continuous variables between groups. Violin plots were used to represent the mean values of the data at the center values, with error bars to indicate s.d. Spearman’s rank correlation tests (two-tailed) were performed to identify significant correlations between two continuous variables. Unadjusted p-value of 0.05 were considered significant for the Mann–Whitney rank sum test and Spearman’s rank correlation tests. Statistical analyses were performed using SPSS V.20.0 for Windows (SPSS, Chicago, IL, USA).

A multivariate statistical analysis was performed using the ropls (Version 1.6.2, http://bioconductor.org/packages/release/bioc/html/ropls.html) R package from Bioconductor on the Majorbio Cloud Platform (https://cloud.majorbio.com). Principal component analysis (PCA) using an unsupervised method was applied to obtain an overview of the metabolic data, and general clusters, trends, or outliers were visualized. All the metabolite variables were scaled to unit variances prior to conducting the PCA. Orthogonal partial least squares discriminate analysis (OPLS-DA) was used as the statistical analysis to determine global metabolic changes between comparable groups. All metabolite variables were Pareto scaled prior to conducting OPLS-DA. The model validity was evaluated from model parameters R2 and Q2, which provide information on the interpretability and predictability, respectively, of the model and avoid the risk of overfitting. Variable importance in the projection (VIP) was calculated in the OPLS-DA model. P-values were estimated with paired Student’s t-test in single-dimensional statistical analyses.

## Results

### SPRC Ameliorated the Symptoms and Inflammatory Response of Arthritis in AIA Rats

**Figure 1:**
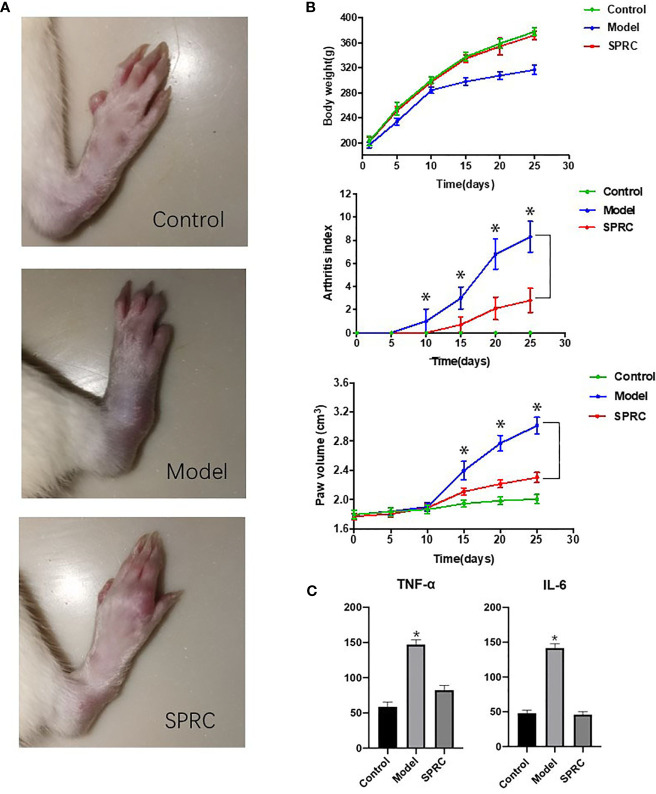
SPRC ameliorated symptoms of arthritis in AIA rats. **(A)** The representative paws of rats in the Control, Model, and SPRC-treated groups. **(B)** Body weight, arthritis index, and paw volume were also recorded, with green lines representing the Control group, blue lines representing the Model group, and red lines representing the SPRC group. The marked asterisks (*) indicated significant differences (p < 0.05) between groups (n=10, means ± SD). **(C)** TNF-α and IL-6 levels were also detected, with asterisks (*) indicating p < 0.05 compared with the Control group (n=10, means ± SD).

The anti-inflammatory effect of SPRC was evaluated on AIA rats, a well-established *in vivo* model of inflammatory joint diseases. As shown in **Figure 1A**, the Model group showed evidently swollen and inflammatory joints, while SPRC treatment ameliorated the severity of these symptoms (28^th^ day). The body weight, arthritis index, and paw volume were recorded every 5 days (**Figure 1B**). The SPRC group showed a significant decrease in both the arthritis index and paw volume compared to the Model group. In addition, lower serum levels of TNF-α and IL-6 were also detected in the SPRC group (**Figure 1C**), suggesting that SPRC treatment effectively ameliorated the symptoms and inflammatory responses of arthritis in AIA rats.

### Gut Microbiota Profile

**Figure 2:**
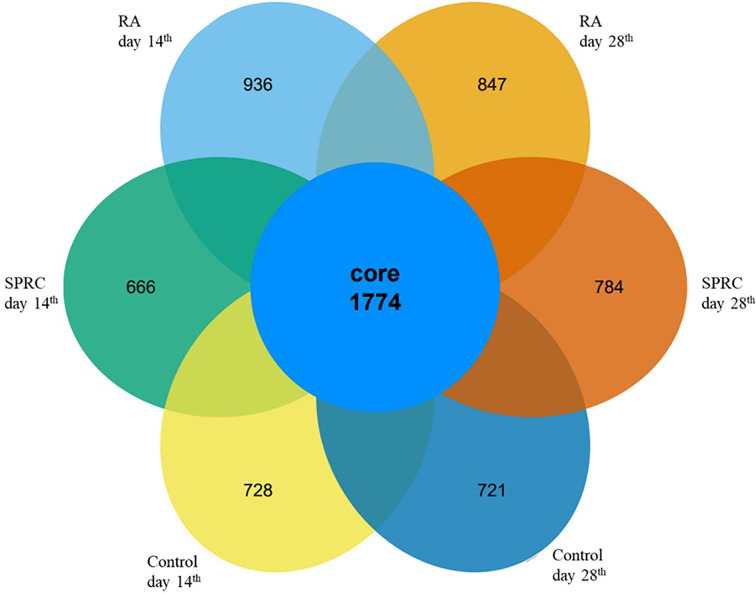
Comparison of the OTUs between groups. The Venn diagram displays the overlaps of 1,774 core OTUs shared among groups at different time points.

The gut microbiota structures of both the Model group and SPRC group were analyzed using 16S ribosomal RNA (rRNA) gene amplicon sequencing. The sequences were grouped into provisional clusters as operational taxonomic units (OTUs). A total of 3,796,465 raw sequences were obtained. After sequence processing, 2,536,086 trimmed reads of 16S rRNA (accounting for 74.9% of the raw sequences) with an average length of 421 bp were obtained. Sequences were classified into species-level OTUs at 97% similarity for each sample. A total of 34,242 OTUs were obtained from all samples, ranging from 486 to 392 OTUs per sample. These OTUs were then categorized into 15 phyla, 28 classes, 52 orders, 105 families, and 200 genera by comparison to a standard database (Silva 119 database). All the samples had a relatively similar complex gut microbiome population and shared 1,774 core OTUs (**Figure 2**). The shared OTUs represented 15, 15, and 18% of the total OTU abundance in the Control, Model, and SPRC groups on the 14^th^ day, respectively, and 18, 17, and 22% of the total OTU abundance in the Control, Model, and SPRC groups on the 28^th^ day, respectively (**Figure 1A**).

### Changes in Biodiversity Were Reversed After SPRC Treatment

**Figure 3:**
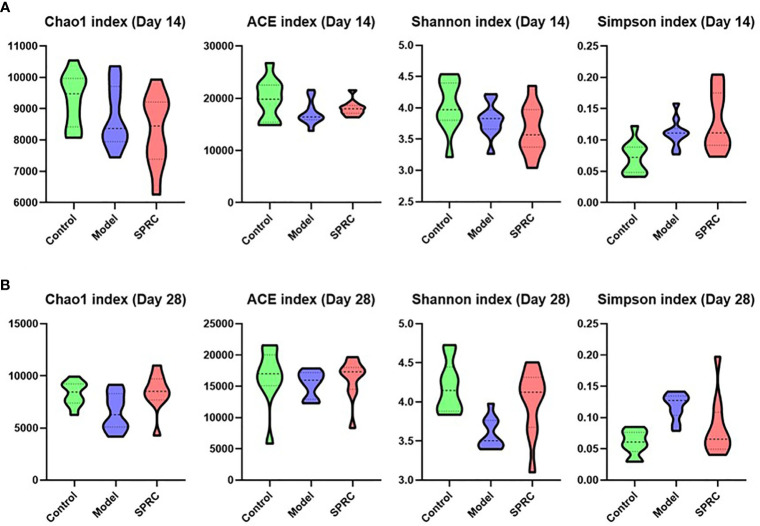
Comparison of the biodiversity between groups. The violin plot outlines illustrate the diversity of gut microbiota on the 14^th^ and 28^th^ days, and the width of the shaded area represents the proportion of the data in each region. **(A)** On the 14^th^ day, compared with other groups, higher stool microbial richness was observed in the Control group, as estimated by Chao1 and ACE indices. Accordingly, microbial diversity in the Control group, as estimated by Shannon and Simpson indices, was also increased. **(B)** On the 28^th^ day, a relatively higher level of microbial richness was observed in the Control and SPRC groups than in the Model group. Accordingly, microbial diversity in the Control and SPRC groups showed a similar trend of higher levels than that in the Model group (n=10).

The richness of bacterial communities in the Model and SPRC groups was estimated by calculating the Chao 1 index and abundance-based coverage (ACE) index, and diversity was estimated by calculating the Shannon diversity index and Simpson diversity index (**Figure 3**). In this study, higher richness was observed in the Control group on both the 14^th^ day and 28^th^ day, as indicated by higher Chao 1 and ACE indices, as well as in the SPRC group on the 28^th^ day. Meanwhile, the diversity assessed by calculating the Shannon and Simpson indices revealed a similar trend of notably higher microbial enrichment on both the 14^th^ and 28^th^ days in the Control group and an increased diversity was observed in the SPRC group on the 28^th^ day.

### Structural Alterations in the Gut Microbiota After SPRC Treatment

**Figure 4:**
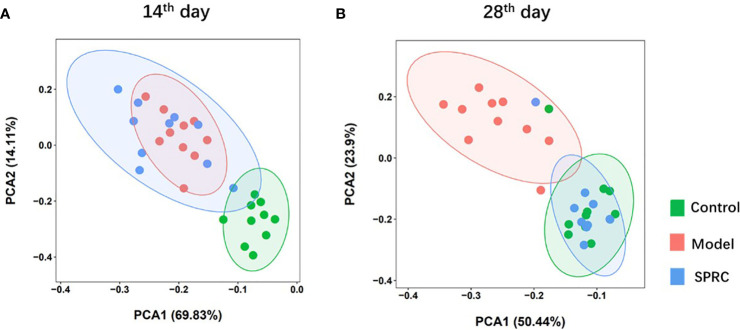
SRPC reshaped the gut microbiota of AIA rats. Principal component analysis (PCA) plot based on the OTU matrix of rat fecal samples from the Control, Model, and SPRC groups on the 14^th^ day **(A)** and 28^th^ day **(B)**.

The overall alterations in the gut microbiota structure at different time points among groups were determined by analyzing the 16S rRNA gene sequences of fecal samples collected from the Control, Model, and SPRC groups. We applied principal component analysis (PCA) to reveal the clustering of gut microbe communities in each group. Notable alterations in the microbiota community structure were induced by SRPC treatments. On the 14^th^ day, the microbiota in the Model and SPRC groups were more closely clustered than that in the Control group, whereas the microbiota in the SPRC and Control groups were more closely clustered than that in the Model group on the 28^th^ day. The changes in the microbiota structure between groups indicated by PCA suggested that SPRC reshaped the gut microbiota during RA treatment (**Figure 4**).

### Key Phylotypes Responding to SPRC Treatment in AIA Rats

**Figure 5:**
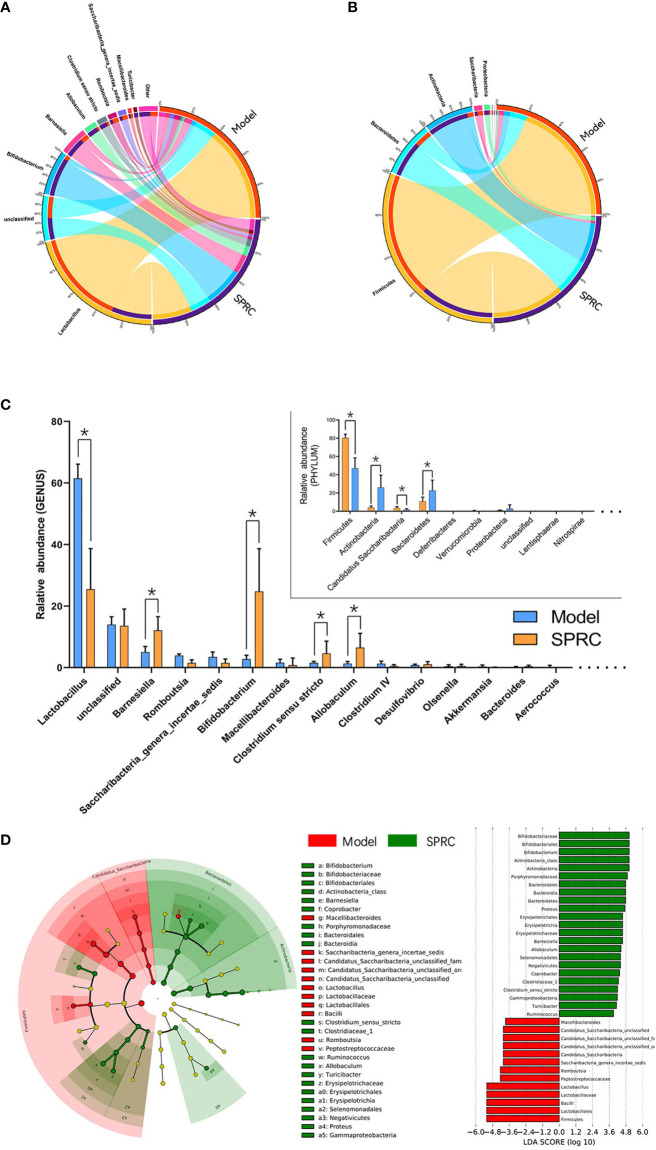
Crucial bacterial candidate selection. The Circos graph shows the relative abundance of microbiota distributions at the phylum **(A)** and genus **(B)** levels between the Model and SPRC groups on the 28^th^ day. The left semicircle represents the phylum composition of each group, and the right semicircle indicates the distribution of each phylum in the different groups. **(C)** Bar plot showing the 10 most abundant phyla and 20 most abundant genera in the Model and SPRC groups, with marked asterisks (*) indicating significant differences (p < 0.05) between groups (n=10, means ± SD). **(D)** Differences in specific taxa between groups were detected using LEfSe, and species with LDA scores higher than 3.6 were plotted. Differences between groups are represented by the color of the most abundant class (red indicates the Model group, and green indicates the SPRC group). The diameter of each circle is proportional to the relative abundance of the microbiota.

The relative abundance of gut microbiota at the phylum (**Figure 5A**) and genus levels (**Figure 5B**) was compared between the Model and SPRC groups on the 28^th^ day. We eventually detected 5 phyla and 28 genera that significantly differed between the Model group and SPRC group according to Student’s t-test. Of the five phyla, *Firmicutes* was the most abundant, accounting for 80.4 and 40.9% of the entire gut microbes in the Model and SPRC groups, respectively. Additionally, four of the five phyla, *Firmicutes*, *Actinobacteria*, *Saccharibacteria*, and *Bacteroidetes*, comprised the main proportion of the total gut microbiota (more than 95%). Moreover, among the 28 genera, *Lactobacillus*, *Bifidobacterium*, *Barnesiella*, *Allobaculum*, and *Clostridium sensu stricto* were dramatically altered between the Model and SPRC groups, with relative abundances greater than 1% in both groups (**Figure 5C**).

Based on species we identified as relevant to the SPRC-treated AIA rats, we used linear discriminant analysis effect size (LEfSe) to further detect the key phylotype, which simplifies the complexity in high-dimensional data while retaining trends and patterns for a series of taxonomic categories. We identified the *Bifidobacterium* genus as the key phylotype enriched in the SPRC-treated group (**Figure 5D**).

### Microbial Metabolic Functions Associated With SPRC Treatment in AIA Rats

**Figure 6:**
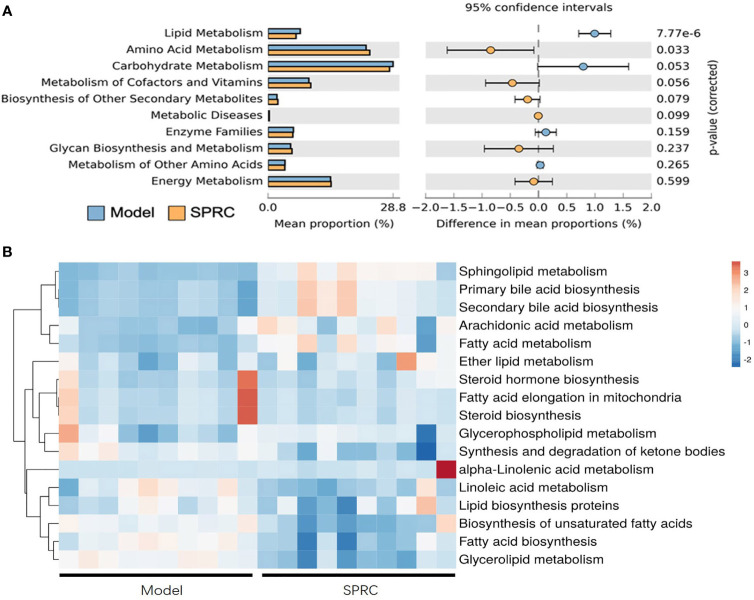
Inferred gut microbiome functions from the PICRUSt analysis of 16S rRNA gene sequences between the Model and SPRC groups on the 28^th^ day. Changes in the functions at KEGG level-2 **(A)** and level-3 **(B)** showed that microbial functions were altered by the SPRC treatment. Colors in the heat map indicate the relative abundance of metabolite levels. Red indicates metabolites that were upregulated, and blue indicates metabolites that were downregulated.

We predicted the functional gene content of bacterial communities by performing a PICRUSt analysis based on the Kyoto Encyclopedia of Genes and Genomes (KEGG) database to characterize the functions encoded by the gut microbiota DNA. Then, we focused metabolism functions on the level-2 KEGG functional classes (**Figure 6A**). Increased functions such as amino acid metabolism, metabolism of cofactors and vitamins, biosynthesis of other secondary metabolites, metabolic diseases, glycan biosynthesis and metabolism, and energy metabolism were observed in the SRPC group. In contrast, the functions of carbohydrate metabolism, enzyme families, metabolism of other amino acids, and lipid metabolism were downregulated in the SPRC group. Additionally, a dramatic decrease in genes related to lipid metabolism was present in the SPRC group (p-value <0.001) compared with the Model group.

Regarding the significantly changed lipid metabolism-related genes, we further detected functional changes in subclasses of this pathway in a level-3 KEGG analysis (**Figure 6B**). Sphingolipid metabolism, primary bile acid biosynthesis, and secondary bile acid biosynthesis were dramatically upregulated in SPRC-treated AIA rats compared with the Model group (*p*<0.001). Moreover, the genes associated with functions of biosynthesis of unsaturated fatty acids, fatty acid biosynthesis, and glycerolipid metabolism were detected at lower levels in the SPRC group. Based on these findings, SPRC treatment showed great potential in reversing gut microbiota functional disorders in AIA rats.

### Overall Metabolomic Analysis of Plasma Samples

**Figure 7:**
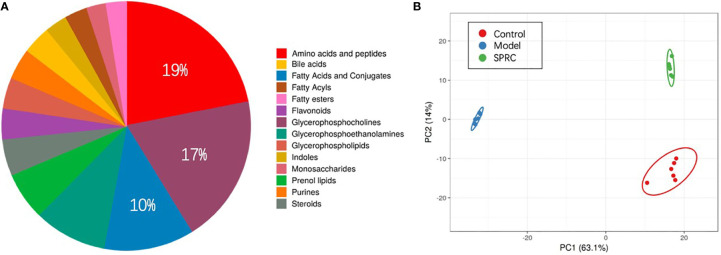
Comparison of the metabolite profiles among groups. **(A)** Pie chart showing the contributions of different metabolite sets in plasma samples. Among all the metabolite sets, amino acids contributed the most (19%), followed by glycerophosphocholine (17%) and fatty acids and conjugates (10%). **(B)** Principal component analysis (PCA) score plot discriminating the metabolic profiles of the three groups. Each dot represents one rat plasma sample, and different colors represent rats from different groups. (R2X =0.77, Q2 = 0.697).

Plasma samples from 18 rats, six in each group, were analyzed by derivatization-UHPLC-Q-TOF/MS. A total of 898 metabolites were identified in plasma samples, and an overview of enriched metabolite sets is shown in **Figure 7A**. The statistical evaluation using principal component analysis (PCA) showed separation of the three groups (**Figure 7B**). In addition, the Model group showed the greatest discrimination in the PCA plot among all three groups.

### Metabolic Profile and Specific Metabolites Altered by SPRC

**Figure 8:**
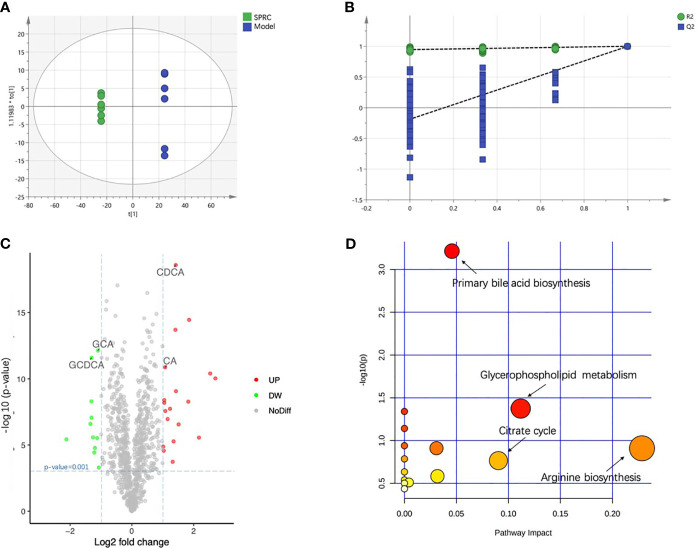
SPRC altered bile acid metabolism. The relative concentrations of all 18 detected metabolites are presented in a heat map. **(A)** The score plot of the OPLS-DA model showed good separation of the Model and SPRC groups. **(B)** A 200-times permutation test of the corresponding model. The Y-axis intercepts were R2 (0, 0.742) and Q2 (0, −0.438). **(C)** The volcano plot visually shows the crucial metabolites marked in green and red with absolute values of a log2-fold change greater than 1 and p-value less than 0.001. The relative concentrations of GCDCA, GCA, CDCA, and CA are also indicated in the plot with arrows. **(D)** Pathway analysis, combining pathway topology analysis and pathway enrichment analysis, annotated the significantly altered metabolites in pathways. The x-axis indicates the pathway impact, and the y-axis represents the pathway enrichment. The nodes with larger sizes and darker colors represent higher pathway impact values and greater pathway enrichment.

A supervised analysis based on the OPLS-DA model was employed to achieve maximum separation between the Model and SPRC groups and to determine the changes in the metabolic profile of AIA rats after SPRC treatment. A clear separation of the Model and SPRC groups is shown in **Figure 8A** (R2X=0.083, R2Y=1, Q2= 0.999), and samples in each group clustered together tightly. Moreover, in the sufficient permutation test, the lines of grouped samples were dramatically located underneath the random sampling lines (**Figure 8B**), indicating sufficient validity for characteristic metabolite identification.

Then, we constructed volcano plots to identify the specifically altered metabolites between groups. As shown in **Figure 8C**, the discrimination between the Model and SPRC groups in the multivariate analysis was mainly linked to the levels of 30 metabolites, 11 downregulated and 19 upregulated, after SPRC treatment. Interestingly, the levels of four metabolites of bile acids contributed substantially to the alterations between groups, including elevated levels of glycochenodeoxycholic acid (GCDCA) and glycocholic acid (GCA) and lower levels of cholic acid (CA) and chenodeoxycholic acid (CDCA).

The significantly altered metabolic pathways impacted by SPRC in plasma were further analyzed among the 30 crucial metabolites. The annotated metabolites were mapped into 15 metabolic pathways according to the Kyoto Encyclopedia of Genes and Genomes (KEGG) database. As shown in **Figure 8D**, arginine biosynthesis, primary bile acid biosynthesis, glycerophospholipid metabolism, and the citrate cycle were the most extensively modulated pathways after SPRC treatment.

### Correlation of the Gut Microbiota and Specific Metabolites

**Figure 9:**
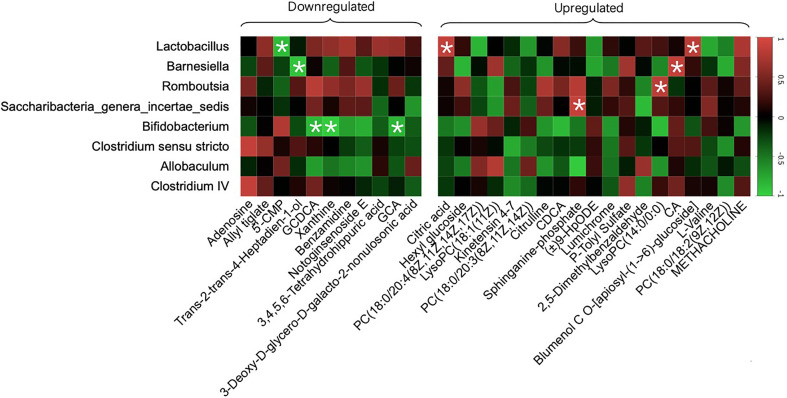
Specific metabolites altered with changes in gut microbiota. The color of each spot in the heat map corresponded to the R value of the Pearson correlation analysis between OTUs of gut microbiota at the genus level and the relative concentration of the metabolites. Spots marked with asterisks (*) represent a significant correlation with |R| <1 and p-value<0.05. The spots in the left matrix represent the downregulated metabolites in the SPRC group; accordingly, the spots in the right matrix represent the upregulated metabolites.

We generated a heat map based on Pearson’s correlation analysis of all 30 crucial metabolites and the gut microbiota with a relative abundance greater than 1% and a p-value<0.05 between the Model and SPRC groups to further explore the relationship between the gut microbiota at the genus level and metabolite changes after SPRC treatment (**Figure 9**). Strong negative correlations were found between *Bifidobacterium* and several specific bile acids, including GCDCA (r=−0.854, p=0.030) and GCA (r=−0.832, p=0.040). Therefore, the decreased level of specific bile acids was closely related to the reshaped gut microbiota structure and increased key phylotypes of *Bifidobacterium* as a result of SPRC treatment in AIA rats.

## Discussion

In the present study, we revealed that the anti-inflammatory effect of SPRC on RA progression was mediated by regulating the gut microbiota. We observed a remarkable shift in the gut microbiota composition of AIA rats after SPRC treatment, and the relevant inflammatory symptoms were also attenuated. Consistent with the functional changes detected in the gut microbiota, bile acid metabolism was regulated in the peripheral blood of SPRC-treated rats. More notably, *Bifidobacterium*, which is one of the genera participating in bile acid metabolism, was found to be significantly increased after SPRC treatment, while the specific metabolites GCA and GCDCA were detected at lower levels in blood.

The dynamic shift of the gut microbiota in individuals with inflammatory diseases has attracted the attention of many researchers in recent years (Chow et al., 2011; Versalovic, 2013). Additionally, alterations in the gut microbiota have also been indicated to be an important driving force in RA, and many hypotheses have been proposed for the underlying mechanism (Drago, 2019; Guo et al., 2019; Rodrigues et al., 2019; Kishikawa et al., 2020). In this study, the gut microbiota was altered significantly and exhibited higher biodiversity, as indicated by the ACE, Chao1, Shannon, and Simpson indices [32], indicating a modulated gut microbiota after SPRC treatment. Moreover, indices reflecting the systemic inflammatory status were measured, and alleviated inflammatory symptoms were found along with lower levels of proinflammatory cytokines, suggesting an attenuated inflammatory response in AIA rats after SPRC treatment (Scher et al., 2015; Kriss et al., 2018; Knox et al., 2019).

In our study, the key phylotype, *Bifidobacterium*, a widely acknowledged probiotic (Heuvelin et al., 2009; Meyer and Vassar, 2018; Achi et al., 2019), was enriched in the SPRC group. *Bifidobacterium* not only exerts anti-inflammatory effects on diseases such as obesity, inflammatory bowel disease, and non-alcoholic fatty liver disease but also represents a potential biomarker of RA progression (Vaahtovuo et al., 2008; Gul’neva and Noskov, 2011). Nevertheless, the relationship between *Bifidobacterium* and RA treatment remains obscure. We used PICRUSt to analyze the functional capabilities of the altered gut microbiota and to determine the possible mechanism of RA treatment in this study. Our results showed a wide range of biological functions that were influenced by SPRC treatment, in which metagenomes were inferred from the 16S data. Primary bile acid synthesis and secondary bile acid synthesis were modulated in the SPRC group, suggesting the role of bile acid-related pathways in RA treatment. Consistent with our results, previous studies also showed that bile acid synthesis is associated with alterations in the gut microbiota and plays a vital role in maintaining glucose, lipid, and energy homeostasis (Li and Apte, 2015; Chen et al., 2019). Moreover, the regulated functions were consistent with the results of our metabolomics study. These findings highlighted the role of abnormal bile acid production by the gut microbiota in provoking RA-related inflammation.

**Figure 10:**
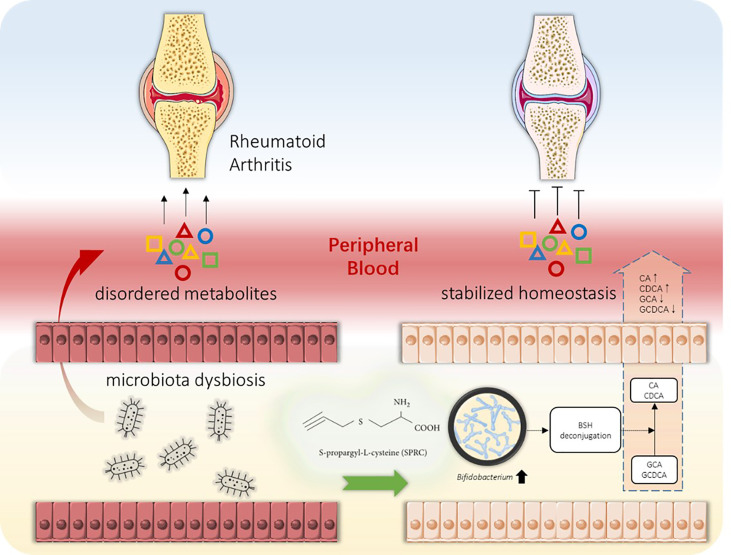
Schematic diagram of the gut-joint axis in RA progression and the intervention strategy. Along with joint inflammatory symptoms, the alteration in the gut microbiota during RA progression might cause metabolic disorders. Abnormal metabolite production provokes peripheral inflammation and enhances the inflammatory response in joint tissue, resulting in pathological synovial tissue inflammation and cartilage impairment (left panel). Oral administration of SPRC remodels the gut microbiota, increases the levels of specific probiotics, reduces peripheral inflammation, and decreases GCA and GCDCA levels, leading to the reversal of RA progression (right panel).

Untargeted metabolomics was applied to plasma samples to further investigate the underlying mechanism. Our results showed that the metabolic profile of the SPRC-treated group was significantly altered compared with that of the Model group. In addition, the *Bifidobacterium* abundance increased dramatically after SPRC treatment and was closely related to the decreased levels of two primary bile acids, GCA and GDCA. BSH has already been identified in several microbial genera, including *Lactobacillus*, *Bifidobacterium*, *Enterococcus*, and *Bacteroide*s. Consistent with our findings, *Bifidobacterium* was found to reduce the levels of GCA and GCDCA by bifidobacterial bile salt hydrolysis (Grill et al., 1995; Kurdi et al., 2006; Sanchez, 2018). Meanwhile, high levels of GCA and GCDCA have been observed in subjects with inflammatory bowel diseases and have close relationships with various inflammatory statuses (Gnewuch et al., 2009; Semba et al., 2017). Moreover, GCA and GDCA have also been reported to induce IL-6 expression, which is an important biomarker of RA progression (Aggarwal et al., 2009). Taken together, our study provides strong evidence for the anti-inflammatory role of the specific bacterium *Bifidobacterium* and key metabolites GCA and GCDCA in SRPC-treated AIA rats, providing new insights into the possible mechanism of SPRC in treating RA (**Figure 10**).

Meanwhile, our study has several limitations. Although we have revealed the mechanism by which SPRC treated RA through alterations in the gut microbiota and metabolites, the anti-inflammatory role of the key bacteria that may be responsible for producing the specific metabolites must be confirmed. Additionally, apart from the key bile acids detected in this study, further exploration will be required to fully understand mechanistic links connecting the gut microbiota with RA-related inflammation.

## Data Availability Statement

The data presented in the study are deposited in the NCBI repository, accession number (BioProject ID: PRJNA744768).

## Ethics Statement

The animal study was reviewed and approved by Animal Care and Use Committee of Municipal Affairs Bureau of Macau.

## Author Contributions

ZW—Study design and literature search. YY—Data collection. JL—Data analysis. HW—Figures. XB—Data interpretation. J-lW—Figures and data interpretation. YZ—Writing. All authors contributed to the article and approved the submitted version.

## Funding

This study was financially supported by the Macau Science and Technology Development fund (FDCT (067/2018/A2 and 033/2017/AMJ, 0007/2019/AKP, 0052/2020/A) and National Natural Science Foundation of China (Grant No. 81973320) provided to YZ.

## Conflict of Interest

The authors declare that the research was conducted in the absence of any commercial or financial relationships that could be construed as a potential conflict of interest.

## Publisher’s Note

All claims expressed in this article are solely those of the authors and do not necessarily represent those of their affiliated organizations, or those of the publisher, the editors and the reviewers. Any product that may be evaluated in this article, or claim that may be made by its manufacturer, is not guaranteed or endorsed by the publisher.
